# Food Enzyme Database (FEDA): a web application gathering information about food enzyme preparations available on the European market

**DOI:** 10.1093/database/baab060

**Published:** 2021-10-09

**Authors:** Marie Deckers, Julien Van Braeckel, Kevin Vanneste, Dieter Deforce, Marie-Alice Fraiture, Nancy h.c Roosens

**Affiliations:** Transversal Activities in Applied Genomics (TAG), Sciensano, Brussels 1050, Belgium; Laboratory of Pharmaceutical Biotechnology, Ghent University, Campus Heymans, Ghent B-9000, Belgium; Transversal Activities in Applied Genomics (TAG), Sciensano, Brussels 1050, Belgium; Transversal Activities in Applied Genomics (TAG), Sciensano, Brussels 1050, Belgium; Laboratory of Pharmaceutical Biotechnology, Ghent University, Campus Heymans, Ghent B-9000, Belgium; Transversal Activities in Applied Genomics (TAG), Sciensano, Brussels 1050, Belgium; Transversal Activities in Applied Genomics (TAG), Sciensano, Brussels 1050, Belgium

## Abstract

Following the European Commission No. 1332/2008 regulation and the consequent necessity of a scientific evaluation of food enzymes (FEs) for their approval for sale on the European Union market, many FE dossiers have been submitted to the European Commission and various documents currently co-exist. In order to centralize all relevant information in one structured location that is easily accessible to support enforcement laboratories and the competent authorities, we developed a web application, called Food Enzyme Database (FEDA). FEDA allows searching and collection of information originating from many different sources in one centralized portal. Queries can be performed using key information types, which include information on the producing company, production source (strain type, genetically modified microorganism status), type of enzyme protein and evaluation status with employed evaluation criteria. The database contains all current publicly available information. Centralizing all information coupled with intuitive searching functionality also allows the generation of general statistics regarding the current market situation. FEDA is open access and is freely available at the following location: https://feda.sciensano.be.

**Database URL** **:** https://feda.sciensano.be

## Introduction

To harmonize regulations and evaluations of food additives, flavorings and enzymes, the European Union (EU) has established a panel of regulations, namely European Commission (EC) Nos 1331/2008, 1332/2008, 1333/2008 and 1334/2008 (1–4). These regulations aim to harmonize evaluation procedures and the subsequent publication of Union lists. Each Union list will, respectively, contain the authorized food additives, flavorings or enzymes allowed to be placed on the European market and used in foods.

However, in contrast to food additives and flavorings (5), no prior European regulations were applicable to food enzymes (FEs). Therefore, all FEs that are or will be commercialized on the European market have to be positively evaluated by the European Food Safety Agency (EFSA) and the EC before being added to the Union list. Applicants were therefore requested to submit dossiers to the EC within a legal deadline, from September 2011 until March 2015, resulting in 303 submitted dossiers (6). These submitted dossiers are mostly confidential but contain a publicly available summary section containing general information. All FE applications submitted within the legal deadline were published on 22 June 2015, associated with a Commission ID and information regarding their production sources and food uses (7).

EFSA first verified the correctness and completeness of the submitted dossiers. Each validated dossier consequently received a specific EFSA ‘question number’, and EFSA is currently performing the scientific evaluations of these dossiers by progressively publishing associated scientific opinions. After evaluation of the 303 FEs by EFSA, the Union list containing all FEs allowed for sale on the EU market will be published by the EC, which is currently expected for 2023. There exists currently a transitory period, during which the available national legislation for FEs are applied for all FE preparations sold on the EU market, which only exists, however, in France and Denmark (8, 9).

**Figure 1.**
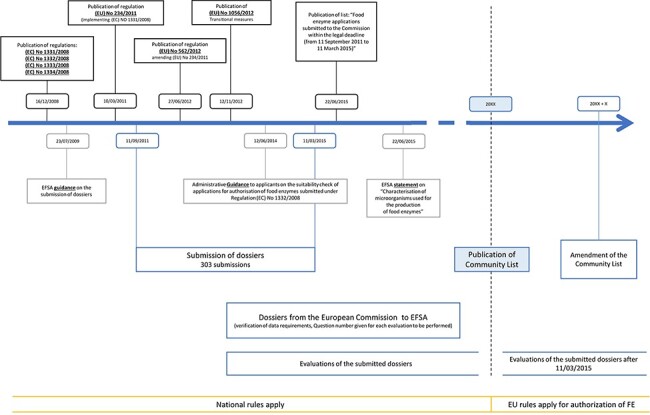
Timeline of the evaluation workflow followed by EFSA for FE preparations following the new harmonization regulations, being 1331/2008, 1332/2008, 1333/2008 and 1334/2008. Additional relevant regulations, statements and amendments are also indicated.

To support the submission procedures of the FE dossiers, several additional regulations, amendments and statements have been published since 2008, as illustrated in Figure 1. First, an EFSA guidance was published on 23 July 2009, clarifying the submission of the dossiers (10). Thereafter, regulation (EU) No. 234/2011 was published (11), containing implementation guidelines for regulation (EC) No. 1331/2008 (1). Subsequently, the amending regulation (EU) No. 562/2012 stated the possibility to submit joint dossiers, combining similar enzyme preparations from various enzyme producers or applicants, resulting in the submission of 16 joint dossiers (12). However, the evaluation of these joint dossiers has proven difficult. Therefore, an ad hoc meeting was held between the EFSA and the Association of Manufacturers and Formulators of Enzyme Products, resulting in partitioning many of these joint dossiers into individual evaluations (13).

Information about transitional measures can be found in regulation (EU) No. 1056/2012 (14), stating that until publication of the Union list, national legislation should be applied to FEs. Guidance for the preparation of new authorization requests or the modification of existing authorizations of FEs can be found in (15). This guideline should be followed for the submission of new requests as of 27 March 2021.

Following the publication of the Union list and new EU legislations on these FEs, there will exist a need for a control system supervising the compliance of FE preparations found on the European market with the appropriate regulations. Therefore, both EU authorities and enforcement laboratories require specific information on these FE for the elaboration of a control plan and the development of appropriate analysis methods.

However, the publicly available information is currently spread over a variety of different documents, such as the public section of the submission dossiers, the list of all submitted dossiers within the legal deadline, and the scientific evaluations. Therefore, searches are currently labor-intensive and can only be performed by targeting specific information in one known document at a time. This dispersion of information in various types of documents complicates searching for information and the possibility for subsequent data analysis allowing to generate global statistics. Moreover, the currently published scientific evaluations exist as PDF documents that do not always adhere to the same structure and contain an increasing amount of confidential data, rendering it difficult to extract specific information. Additionally, the use of different reference codes by the EC and EFSA for the same submitted FE dossiers complicates managing the available information about FEs.

To simplify consulting, collecting and analyzing FE data, we have collected all publicly available information about FEs that can be sold on the EU market from which key information was extracted, harmonized and structured. This information was centralized in a newly developed web application, named Food Enzyme Database (FEDA), to support the competent authorities and enforcement laboratories. In particular, this web application provides a search engine where both general information, such as producing organisms, genetically modified microorganism (GMM) status, producing company, etc., and specific information (related to a specific enzyme dossier) can be used as filtering criteria. FEDA facilitates the searching of all relevant information compiled from various sources in one central location. The compiled data additionally allows the provision of statistics concerning the current products found on the EU market.

## Data collection

The elements presented in Table 1 were selected as key information from the publicly available data. For each type of key information, a description can be found as well as the corresponding reference to the document(s) needed to gather this type of information.

**Table 1.** Identification of types of key information and their description.

Type of key information	Definition	Reference
Enzyme protein	An enzyme protein is a pure enzyme, which is used in laboratories.	(7)
Food enzyme	A food enzyme is the result of the fermentation or extraction process and the subsequent downstream processing steps. An FE contains one or multiple active enzyme protein(s) as well as by-products of the fermentation or extraction method.	(7)
Commission ID	The EC allocated a reference number to each submitted FE dossier.	(7)
EFSA question number	For each evaluation that needs to be performed, an EFSA question number was allocated. In case multiple enzyme proteins are present in the FE preparation, one Commission ID corresponds to multiple question numbers.	(7, 16)
Producing organism	An FE can be produced by different sources, by fermentation using microbial species or by extraction from plants or animals. Additionally, the strain was identified and whether or not the strain has genetically been modified.	(7)
Evaluation status	This provides information on whether a dossier has been withdrawn, rejected for evaluation or already been evaluated by EFSA.	(16)
Submitter	The submitting company/association of the corresponding FE dossier.	(16)
Food uses	This represents the various application fields for which the corresponding FE can be used.	(7)

The reference sources to the corresponding document(s) from which the information was collected are also provided.

The data for the web application has been collected from various publicly available sources, such as the public parts of the submitted FE dossiers, the EFSA portals (16, 17), published scientific opinions, the NCBI databases (genomes and taxonomy), EC inventory and International Union of Biochemistry and Molecular Biology (IUBMB) nomenclature.

First, the list of all dossiers submitted within the legal deadline was analyzed to identify all FEs that will be evaluated and their application uses. This allowed the identification of FEs that could be present on the European market after the publication of the Union list. Additionally, all the producing organisms mentioned for FE production were identified, resulting in a list of microbial species, plants and animals.

One of the purity criteria analyzed during the scientific evaluation of an FE is the absence of the production strain in terms of both viable cells and associated DNA. Therefore, besides the name of the microbial species (as mentioned in the submitted dossiers), the strain number and genetically modified (GM) status were also collected. Subsequently, information on the submitting companies was retrieved from the public sections of the submitted dossiers.

Using the EFSA register of questions, all requests for safety evaluations were extracted with their corresponding EFSA Question numbers. At the moment of data collection, no document existed associating the commission ID (from the submission dossiers) with the EFSA Question numbers. Therefore, we cross-linked the available information to provide this association in the web application, the information from the public sections of the submission dossiers to be connected with their evaluation status and the already published scientific opinions. Afterward EFSA published a register providing a link between the two codes (18). Additionally, the EFSA portal allowed the identification of the dossiers that have been withdrawn or not accepted for evaluation.

Besides the information from the EFSA and EC documents, additional general information was collected. The following information was gathered for all identified enzyme proteins: systematic name, IUBMB number and nomenclature, European Commission (EC) number, Chemical Abstract Service Registry number, synonyms and the properties of the enzyme action.

For all production sources (microbials, plants or animals), information about their genome size and taxonomy ID was collected, when available, from NCBI. For the microbial sources, additional information was collected about their optimal culturing conditions, medium, temperature and time. This information was obtained from our own collected reference strains, used in previous publications for method development and testing (19, 20).

Last, from the published scientific opinions, information regarding the purity criteria was extracted. The different analyzed impurities by EFSA during the scientific evaluations were identified as well as their corresponding results and employed limits of detection. Besides tested impurities, the following information was also extracted from the scientific opinions; the enzyme’s food uses, intended enzyme use levels, manufacturing process, downstream processing and enzyme activity. For the data verification, the four eyes principle was applied, i.e. after collecting and structuring the identified data, a verification was performed by a second scientist to guarantee its correct collection and representation.

## FEDA database construction

### Development

The FEDA web application back-end is developed in Python 3.6. The front-end is written in HTML and JavaScript, using JQuery https://jquery.com/ and Bootstrap libraries https://getbootstrap.com/. Furthermore, the whole application is developed using the web2py framework https://github.com/web2py/. On the infrastructure side, the web interface is served by uWSGI https://uwsgi-docs.readthedocs.io/en/latest/ behind an Nginx proxy https://nginx.org/en/. Installation to a dedicated Ubuntu 18.04 server is automated through Ansible https://www.ansible.com/. The database itself is stored inside an internal PostgreSQL https://www.postgresql.org/ server. For exporting reports, the application’s html to pdf conversion feature uses the textline (TeX) distribution https://tug.org/texlive/ to generate PDF documents on the fly. FEDA is optimized for Google Chrome and Mozilla Firefox and can be accessed online, without requiring registration, at the following location: https://feda.sciensano.be.

### Structure of the application data model

**Figure 2.**
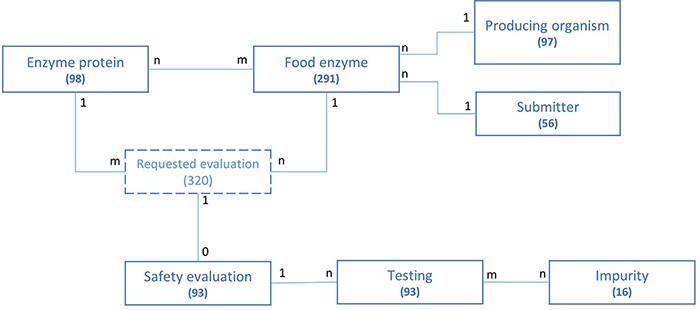
Schematic representation of the underlying data model structure of the web application, representing the relationships between the different model objects. For each entity, the total number of corresponding entries in the database is given (valid at the time of submission). The numbers at each association indicate cardinalities.

The structure of the FEDA application data model and the association between its different entities are represented in Figure 2 in a simplified model. For each entity, the number of collected and integrated entries is given. The FE serves as the main entry point of the application and is the central object connected to all others. Most entities are bound by a ‘one to many’ relationship. For instance, an FE is produced by one organism, while the same producing organism can be used to manufacture various FEs. The same type of relationship exists between the submitter and the FE. Some entities share a more complex ‘many to many’ relationship. This is, for instance, the case for the enzyme proteins and FEs: an FE can contain multiple enzyme proteins and an enzyme protein can be part of a multiple FE. One or multiple safety evaluations could have been requested for one FE, depending on the amount of enzymes present in the FE, while a safety evaluation is linked to only one food enzyme. An FE containing multiple enzyme proteins will therefore be linked to multiple evaluations. The abstract entity ‘Requested evaluation’ allows connecting the enzyme protein(s) present in a food enzyme to the safety evaluation performed by EFSA. It is only used for modeling purposes and does not reflect the underlying structure of the application nor any business key information.

A full database business scheme can be found in Supplementary file 1, providing a more detailed representation of the underlying database structure of the web application. All provided results and statistics in this manuscript were valid at the time of submission.

### Particular cases during data integration

In some exceptional cases, alterations of database entries had to be made in order to harmonize the data. First, enzyme proteins are allocated an IUBMB number. However, for some enzyme groups for which FE dossiers were submitted to EFSA, no such number applies. This is, for instance, the case for ‘rennet’, which is a mixture of enzymes obtained from the stomach of different animals that can contain chymosin, pepsin and lipase. Since the term ‘rennet’ is used for a mixture of enzymes, no corresponding IUBMB number is available. In this case, the number 0.0.0.0 is used within the database. The same applies for the enzymes pancreatin and plant coagulant. For enzymes representing a certain group of enzymes, the missing numbers were completed by a zero. This was the case for protease (3.4.0.0) and d-fructose 4-epimerase (5.3.3.0). Second, from the published scientific opinions, information about the enzyme level of use and the exposure level was extracted. For some enzymes, a range of values is provided. In those cases, the highest level was introduced within the database. Last, for some enzymes, an extension of use (i.e. the addition of food uses) was added to the primary described usage of the enzyme application. In this case, a second EFSA Question number was generated, which was also separately incorporated into the database.

## FEDA interface

The FEDA interface provides a search engine allowing the use of key information types as filtering criteria to restrict the scope and navigate within the database (Supplementary file 2A and B). Searches can be performed based on the following criteria: protein name, submitter, Commission ID, production source, application, production method and GMM status. It should be noted that searches in FEDA need to be performed using the reference name of the enzymes. The ‘synonyms’ dictionary, available from the search page, can be used to verify the enzyme terminology that needs to be used in case other commonly used names exist.

After searching, an overview table of the matching database entries is generated, displaying detailed information for every listed FE dossier (Supplementary file 2C). This overview table can be exported as a comma-separated value or tab-separated value file.

Inside the search results, each line contains a link with more information about the selected FE (Supplementary file 2D). FE pages constitute the starting point for navigation, linking to additional information about, amongst others, the producing organism (Supplementary file 2E), the enzyme protein(s) (Supplementary file 2F) and the scientific evaluation in case this has already been published by EFSA (Supplementary file 2G). Additionally, the public part of the submitted FE dossier can be exported.

After completion of the scientific evaluation by EFSA, an overview of the obtained results and conclusions are also represented and the EFSA scientific opinion file can also be exported. For all previously described types of information, PDF exports can be made. An illustrated user manual, describing how to use the different pages of the web application, can also be accessed at any moment by navigating to the ‘Help’ option in the top menu displayed in every application page.

Regular maintenance and updates, adding information of future enzyme dossiers, of the database will be performed. Recent updates to the database are displayed and listed on the application home page.

## Applicability of FEDA: research questions

To illustrate potential applications of FEDA, some research questions mimicking different situations that enforcement laboratories and the competent authorities could encounter were elaborated and provided in the following sections.

First, both specific information and general information types can be collected, based on how specific searches are executed. Second, FEDA can be used to generate relevant general statistics related to global analysis of FEs.

### Individual examples of application

#### What are the producing organism(s) of a specific FE?

**Figure 3.**
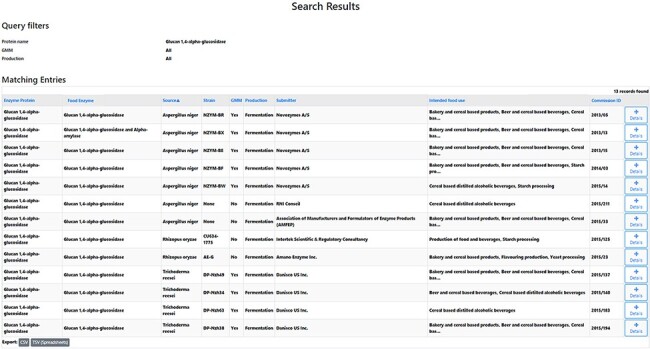
Search results research question 5.1. A list of 13 records is obtained. The table headers can be used to filter the obtained results. By selecting the ‘Details’ icon, the user is redirected to the detailed information of the selected FE. From those pages, amongst others, the EFSA status can be collected.

After experimentally detecting a certain microorganism in a tested FE preparation, it can be relevant to ascertain whether this microbial species corresponds to the producing organism of this FE. One specific question could for instance be: What are the producing organisms of glucan 1,4-alpha-glucosidase preparations? An FEDA search using glucan 1,4-alpha-glucosidase (glucoamylase) as the protein name resulted in a list of 13 obtained records (Figure 3, Supplementary file 3). Each line contains information on the FE, the enzyme protein, its corresponding producing organism and strain, GMM status, the production process, the submitter of the FE dossier, its food use(s) and the Commission ID. The results indicate that this enzyme is only produced by fungal sources from three species: *Aspergillus niger, Rhizpous oryzae* and *Trichoderma reesei*. Consequently, in this case, a detected bacterial strain will likely not be the producer organism of the FE.

#### Has a specific FE preparation been evaluated by EFSA?

A more in-depth analysis of the previously obtained records under the ‘What are the producing organism(s) of a specific FE’ section enables the gathering of more information about their current EFSA safety evaluation status. This can be achieved by accessing each individual record by clicking on their respective ‘Details’ icon (Supplementary file 4). Each entry will be opened in a separate tab, allowing the user to collect the additional information about each evaluation status. Using the previous example of glucan 1,4-alpha-glucosidase FE preparations, by inspecting the individual 13 FE pages, it can be observed that currently four FEs have positively been evaluated by EFSA. Additionally, for two of the four FEs produced by *T. reesei*, the submitted dossier has been withdrawn and will therefore not be evaluated and one of the FEs produced by *A. niger* has received a negative evaluation by EFSA. For the remaining Fes, the evaluation process is still ongoing.

### Obtaining general statistics on FE intended for the European market

#### Are FEs often produced by GMMs?

FEDA can also be used to generate relevant general statistics about the current EU FE landscape by combining different searches. For instance, one relevant question would be the percentage of FEs found on the EU market produced by GMMs. By performing a search in FEDA selecting only the production by a GMM as a criterion, a list of all enzyme proteins produced by GMMs for which a dossier was submitted for evaluation can be generated. This results in 130 records (Table 2). Using column sorting based on the FE name, both alpha-amylase (12.3%) and xylanase (12.3%) are currently found to be the enzymes most frequently produced by GMMs. For both enzymes, a total of 16 records are currently present in the database. By performing an unspecified open search, the total number of enzyme protein entries present in the database can be obtained, resulting in a total of 320 records. This results in 130 out of the 320 entries (40.6%) of enzyme proteins being produced by a GMMs. The total number of GMM entries can also be determined in comparison with only the microbial entries. Therefore, the ‘fermentation’ option should be selected, resulting in a total of 284 entries. Consequently, we can determine that 88.8% (284/320) of all entries are produced by a microorganism and that 45.8% (130/284) of all microbial entries use a GMM strain for the production of the enzyme protein.

**Table 2.** Examples of relevant statistics that can be generated with FEDA

Information type	Status on time of submission	Statistics
Total entries	320	/
Microbial FE entries	284	88.8% ^a^ (284/320)
Microbial GMM entries	130	40.6% ^b^ (130/320) 45.8% ^c^ (130/284)
Total enzyme proteins	98	/
Total producer organisms	97	/
Total submitters^e^	56	/
GM microbial species^e^	20	20.6% ^d^ (20/97)

a The percentage of microbial FE entries is determined in relation to the total number of entries in the database (containing microbial, plant and animal enzymes).

b The percentage of microbial GMM entries is determined in relation to the total number of entries in the database (containing microbial, plant and animal enzymes).

c The percentage of microbial GMM entries determined in relation to the number of microbial entries in the database (excluding plant and animal FE entries).

d The percentage of GM microbial species determined by combining the 97 different microbial producer organisms integrated in the database with the 20 microbial species for which a GMM strain was entered.

e Data generated by exporting the database results page and analyzing the data in Excel.

Such percentages can also be determined for other information types. A few additional examples are given in Table 2 and hereunder.

The total number of different enzyme proteins and the total number of different producer organisms can be collected by using the dropdown lists available from the question marks on the search page, resulting in 98 different enzyme proteins and 97 producer organisms By exporting the results pages (by selecting the tab-separated value or comma-separated value option at the bottom of the page), additional information can be generated using simple Excel analysis exploration (such as pivot tables). Doing this, one can, for instance, observe that dossiers were submitted by a total of 56 different submitters. The export of the search results from only the 130 GMM entries shows that 20 different species are mentioned as GM producer organisms Compared with the total of 97 different producer organisms mentioned in the database, it can be deduced that 20.6% (20/97) of the FE producer organisms mentioned in the database include GMM strains.

#### At what level are enzymes, produced by GMMs, used in a specific food process?

It is also possible to collect information about the intended level of use of specific FEs based on their published EFSA safety evaluations. In case no scientific opinion has yet been published for an FE preparation, information can be collected from similar products even when no prior information is known about these other preparations. This information can help the competent authorities to evaluate potential health risks in case impurities would be detected, related to the ingestion of this preparation. For instance, one potential question could be the intended level of use for FE preparations produced by a GMM used for dairy processing. A specific search looking for all enzyme preparations produced by GMMs and used for dairy processing resulted in 14 records, for which the intended level of use can be accessed on each detailed record page. With the collected information of this specific case, we can find that the enzyme beta-galactosidase, produced by a GMM *Escherichia coli,* is used for dairy processing at a level of 200 mg TOS/kg lactose (total organic solids). Even though this information might currently only be available for one actual record, it can be used as a proxy for other GMMs *E. coli* beta-galactosidase for dairy processing until this information is publicly available after evaluation of the remaining dossiers by EFSA.

## Conclusion

Since information on FE preparations and their safety evaluations are dispersed across many different documents and portals, there exists a need to centralize all relevant information in one structured location that is easily accessible to support enforcement laboratories and the competent authorities. We have developed a web application, FEDA, storing all publicly available information in one centralized portal, which allows users to search and collect information originating from many different sources. This will facilitate searching for information when only limited prior information is known. Centralizing all information coupled with intuitive searching functionality allows users to generate general statistics about the current market situation. FEDA can be accessed online at the following location: https://feda.sciensano.be. FEDA is optimized for Google Chrome and Mozilla Firefox and is completely open access without requiring registration.

Various key information types were identified, for which publicly available data was collected and incorporated into the FEDA database. These information types include, among others, information on the producing company, the producing source (strain type and GMM status), the type of enzyme protein and the evaluation status with employed evaluation criteria. The collected data contains all currently available public data. Confidential data, including information on genetic modifications in case the producing organisms were genetically modified, could however not be integrated.

A search engine allows the user to collect data of interest in a simple and intuitive fashion. Additionally, FEDA allows the user to connect information from the submitted FE dossiers with their scientific evaluations, since different reference codes are given to both.

As we are currently in a transitional period, various types of FEs co-exist on the EU market. There are FEs for which a dossier was submitted, which will be evaluated for adding to the Union list of authorized FEs, but there exist also FEs for which no dossier was submitted, which will therefore not be allowed on the EU market once the Union list will be published. FEDA currently contains all publicly available information, related to 291 FEs including 93 evaluated FEs. Frequent maintenance and updates, adding information from newly published scientific evaluations, will be performed. Once the Union list will be available and consequent regulations will be enforced, FEDA will represent the European FE market, providing users a complete overview of all EU-authorized FEs.

## Supplementary Material

baab060_Supp

## References

[1] The European Parliament and the Council of the European Union (2008) Regulation (EC) No 1331/2008 of the European Parliament and of the Council of 16 December 2008 establishing a common authorisation procedure for food additives, food enzymes and food flavourings. *Off. J. Eur. Union*, 354, 16–33.

[2] The European Parliament and the Council of the European Union (2008) Regulation (EC) No 1332/2008 of the European Parliament and of the Council of 16 December 2008 on food enzymes and amending Council Directive 83/417/EEC, Council Regulation (EC) No 1493/1999, Directive 2000/13/EC, Council Directive 2001/112/EC and(.). *Off. J. Eur. Union*, 51, 7–15.

[3] The European Parliament and the Council of the European Union (2008) Regulation (EC) No 1333/2008 of the European Parliament ans of the Council of 16 December 2998 on food additives. *Off. J. Eur. Union*, L 354, 16–33.

[4] The European Parliament and the Council of the European Union (2008) Regulation (EC) No 1334/2008 of the European Parliament and OF the Council of 16 December 2008 on flavourings and certain food ingredients with flavouring properties for use in and on foods and amending Council Regulation (EEC) No 1601/91, Regulations (EC). *Off. J. Eur. Union*, 1334, 34–50.

[5] Commission Regulation (2012) (EU) No 231/2012 of 9 March 2012 laying down specifications for food additives listed in Annexes II and III to Regulation (EC) No 1333/2008 of the European Parliament and of the Council.

[6] European Food Safety Authority (2014) Administrative Guidance to applicants on the suitability check of applications for authorisation of food enzymes submitted under Regulation (EC) No 1332/2008. *EFSA Support. Publ.*, EN-638, 17.

[7] European Commission (2016) Food enzyme applications submitted to the Commission within the legal deadline (from 11 September 2011 to 11 March 2015). July 2016.

[8] (2020) Bekendtgørelse om tilsætninger mv. til fødevarer 1).

[9] Cerutti G. , Boudot J., Bournigal J.-M. et al. (2006) Arrêté du 19 octobre 2006 relatif à l’emploi d’auxiliaires technologiques dans la fabrication de certaines denrées alimentaires. [Online]. https://www.legifrance.gouv.fr/affichTexte.do?cidTexte=LEGITEXT000020667468#LEGISCTA000020667473 (11 May 2021, date last accessed).

[10] Anadón A. , Bell D., Binderup M.L. et al. (2009) Guidance of the scientific panel of food contact materials, enzymes, flavourings and processing Aids (CEF) on the submission of a dossier on food enzymes for safety evaluation by the scientific panel of food contact material, enzymes, flavourings and proc. *EFSA J.*, 1305, 1–26.

[11] The European Commission (2011) Commission Regulation (EU) No 234/2011 of 10 March 2011 implementing Regulation (EC) No 1331/2008 of the European Parliament and of the Council establishing a common authorisation procedure for food additives, food enzymes and food flavourings. *Off. J. Eur. Union*, 2011, 15–24.

[12] The European Commission (2012) Commission Implementing Regulation (EU) No 562/2012 of 27 June 2012 amending Commission Regulation (EU) No 234/2011 with regard to specific data required for risk assessment of food enzymes. *Off. J. Eur. Union*, 168, 21–23.

[13] European Food Safety Authority Ad hoc meeting with industry representatives – joint dossiers on food enzymes produced from animal and plant sources. 18 September 2020. [Online]. https://www.efsa.europa.eu/en/events/event/ad-hoc-meeting-industry-representatives-joint-dossiers-food-enzymes (22 December 2020, date last accessed).

[14] The European Commission (2012) Commission Regulation (EU) No 1056/2012 of 12 November 2012 amending Regulation (EC) No 1332/2008 of the European Parliament and of the Council on food enzymes with regard to transitional measures. *Off. J. Eur. Union*, 313, 9–10.

[15] EFSA (European Food Safety Authority) (2021) Administrative guidance for the preparation of applications on food improvement agents (food enzymes, food additives and food flavourings). *EFSA Support. Publ.*, 18, 37.

[16] European Food Safety Authority (2010) EFSA Register of Questions. [Online]. https://registerofquestions.efsa.europa.eu/roqFrontend/login?0 (01 June 2021, date last accessed).

[17] European Food Safety Authority OpenEFSA portal. [Online]. https://open.efsa.europa.eu/ (01 June 2021, date last accessed).

[18] European Commission (Directorate-general for health and food safety) (2020) Register of food enzymes to be considered for inclusion in. 97, 21.

[19] Deckers M. , Vanneste K., Winand R. et al. (2020) Strategy for the identification of micro-organisms producing food and feed products: bacteria producing food enzymes as study case. *Food Chem.*, 305, 125431. 10.1016/j.foodchem.2019.125431 31610425

[20] Deckers M. , Vanneste K., Winand R. et al. (2020) Screening strategy targeting the presence of food enzyme-producing fungi in food enzyme preparations. *Food Control*, 117, 107295.

