# Correction to: FEDA (Food Enzyme Database): a web application gathering information about food enzyme preparations available on the European market

**DOI:** 10.1093/database/baac012

**Published:** 2022-03-19

**Authors:** Marie Deckers, Julien Van Braekel, Kevin Vanneste, Dieter Deforce, Marie-Alice Fraiture, Nancy H C Roosens

**Affiliations:** Transversal Activities in Applied Genomics (TAG), Sciensano, Brussels 1050, Belgium; Laboratory of Pharmaceutical Biotechnology, Ghent University, Campus Heymans, Ghent B-9000, Ghent, Belgium; Transversal Activities in Applied Genomics (TAG), Sciensano, Brussels 1050, Belgium; Transversal Activities in Applied Genomics (TAG), Sciensano, Brussels 1050, Belgium; Laboratory of Pharmaceutical Biotechnology, Ghent University, Campus Heymans, Ghent B-9000, Ghent, Belgium; Transversal Activities in Applied Genomics (TAG), Sciensano, Brussels 1050, Belgium

In the originally published version of this manuscript, there was a typographical error in the second author’s name. This should read: ‘Julien Van Braekel’ instead of ‘Julien Van Braeckel’. This error is now corrected online.

