# Supplementary material for: Food Enzyme Database (FEDA): a web application gathering information about food enzyme preparations available on the European market
Source: Database (Oxford). 2021 Oct 9;2021:baab060. doi: 10.1093/database/baab060 (PMC8502015; doi:10.1093/database/baab060)
Supplement: baab060_Supp [file baab060_supp.zip › Supplementary files-final...docx]

**Supplementary File 1: Database scheme**

The database scheme, provides a more detailed representation of the underlying database structure of the web application. Within each table, the first column represents the field name and the second column the field type or function. Each table has its unique and mandatory identifier, the ID or primary key (pk). Between the different tables, the relationships are shown by either straight or dotted lines together with the type of relation, indicated with 1, n or m for respectively one to one, one to many, or many to many relationships. The dotted lines are used in case an intermediate table is used. ‘Foreign keys’ (fk) refers to the ‘primary keys’ of another table, representing relationships between tables. Within the tables the information fields are preceded by a filled or empty circle, the former indicates the corresponding field is mandatory, the latter indicates the field is non-mandatory. Different field types are used: Boolean, floating point, integer text, enumerate, datetime char or varchar. In cases of var and varchar (storing characters), the limit of characters is also indicated between brackets. Files, such as PDFs of published EFSA scientific opinions, can be uploaded through the application and stored under the ‘file’ field type. Lastly, besides the single value fields, lists are used for integration of multiple values in the same table field.


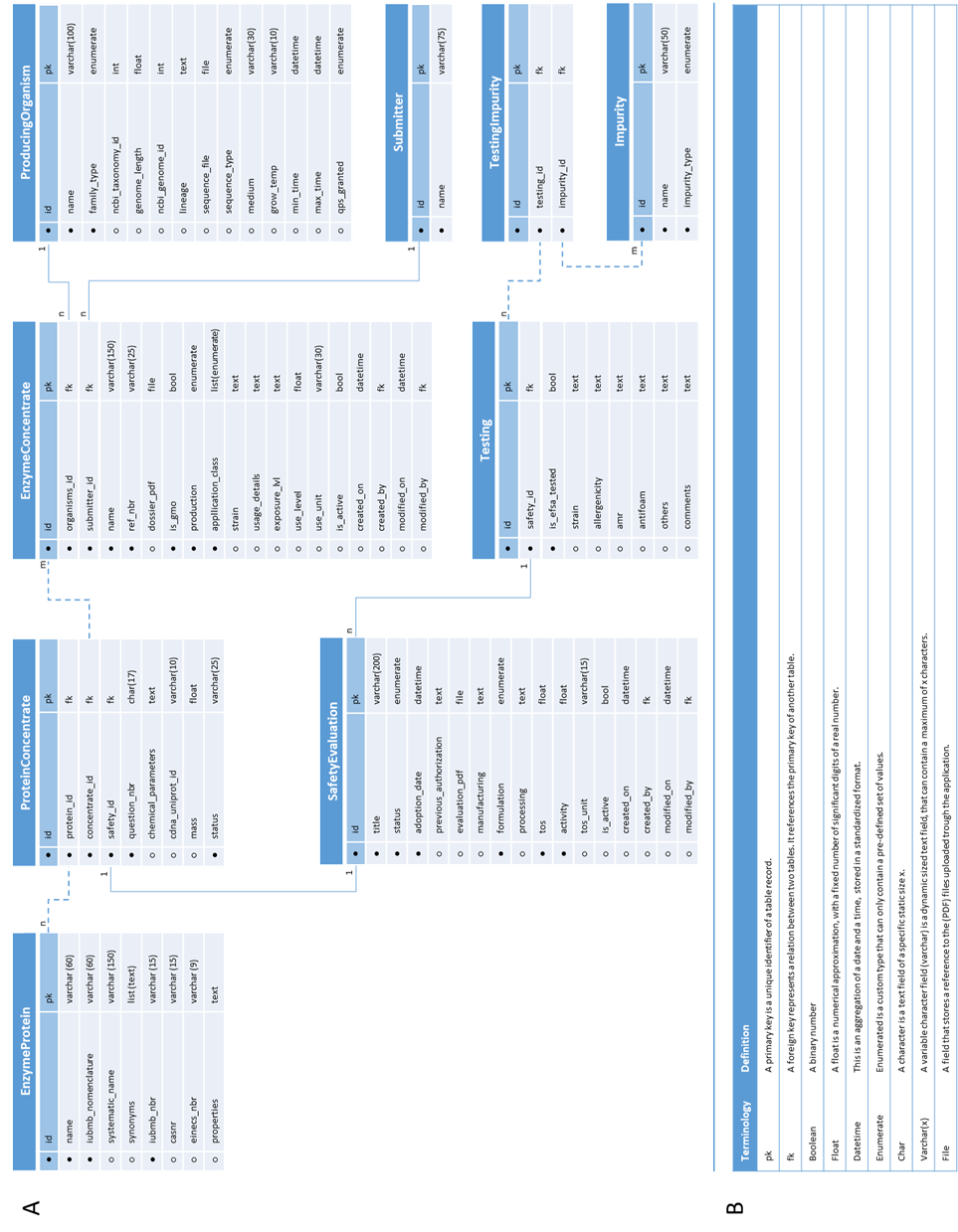


*Figure 1: A: Database business scheme representing the application tables, their respective fields and relationships. Additional, unrepresented tables are present for the database change tracking feature and the user management system.*

*B: Definitions are provided to explain the differ types of fields.*

**Supplementary File 2: Use and access of the FEDA (Food Enzyme Database)**


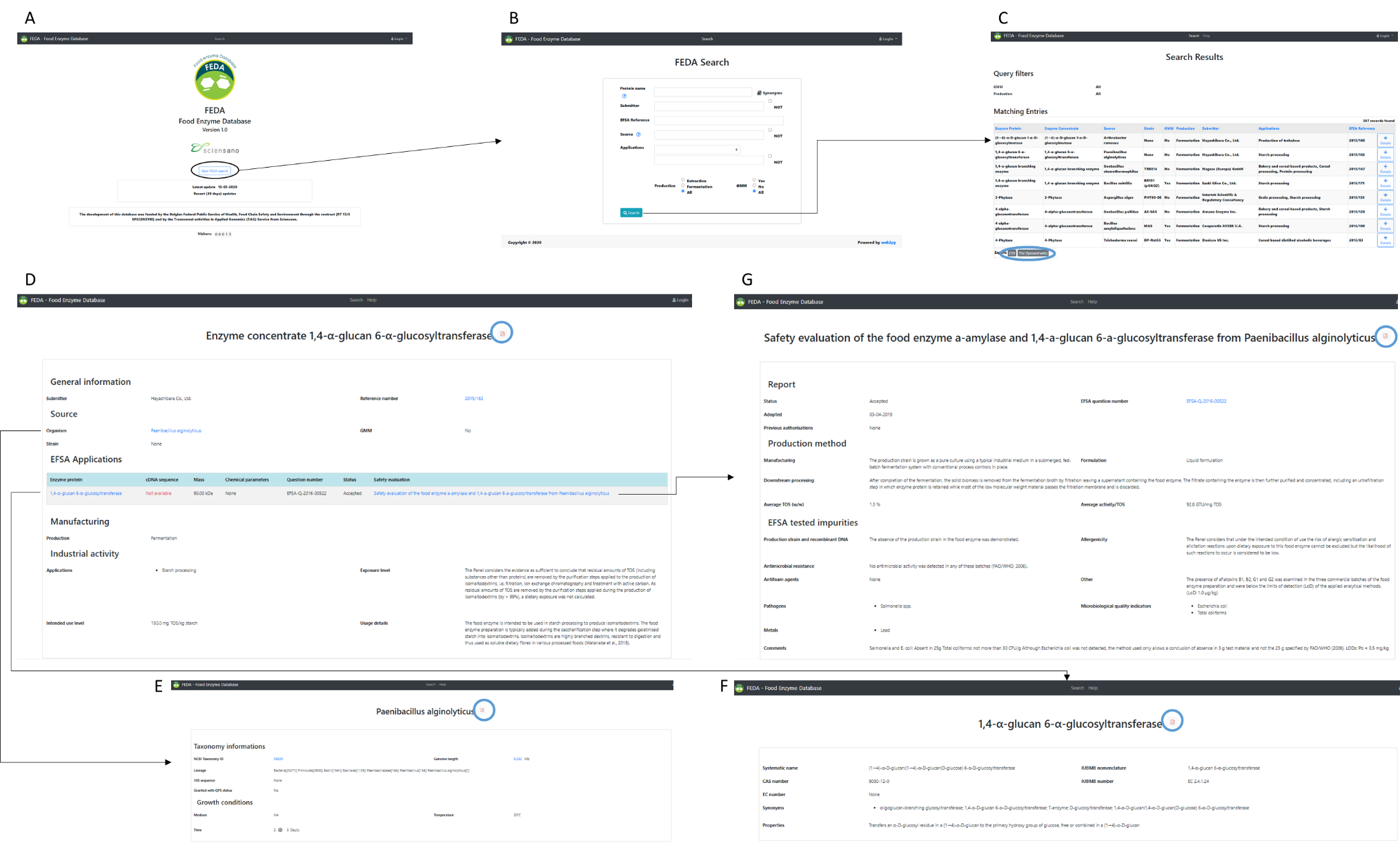


*Figure 2: Representation of the database usage. From the start page (A), the search engine can be accessed (B). After selection of the desired search criteria, a results table is obtained (C). From there, specific information about an enzyme concentrate (D) can be obtained by selecting the “detail” button after the desired row. Additional information about the producing organism (E), the enzyme protein (F) and the safety evolution (G) can be obtained from this concentrate page.*

*The different export options are indicated with the blue circles.*

**Supplementary File 3: Research question 1 workflow**


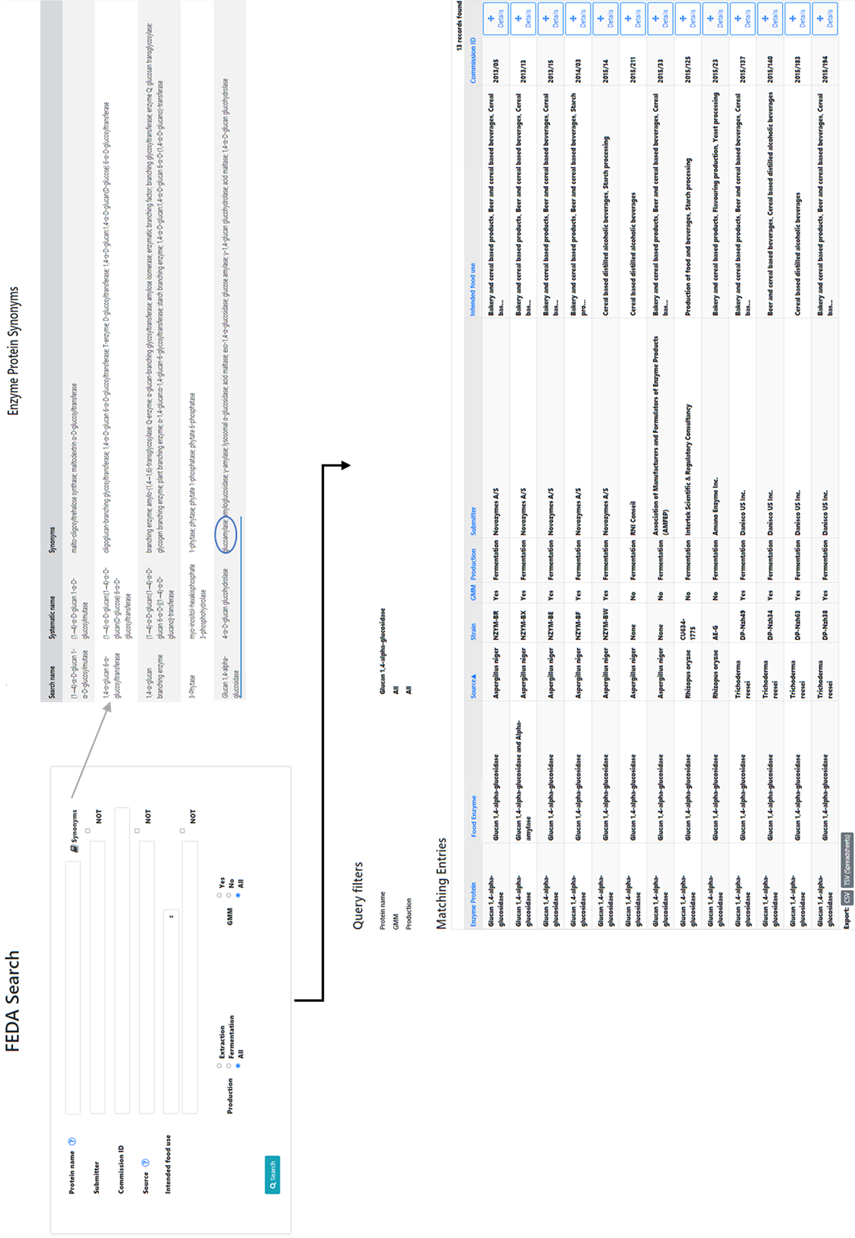


*Figure 3: Representation of the steps to follow in order to find the results of the study case 1 (What are the producing organisms of Glucan 1,4-alpha-glucosidase preparations?). A search is performed on the enzyme protein. A results overview is generated, allowing the users to access detailed information for each food enzyme. This table can be exported.*

**Supplementary File 4: Research question 2 workflow**


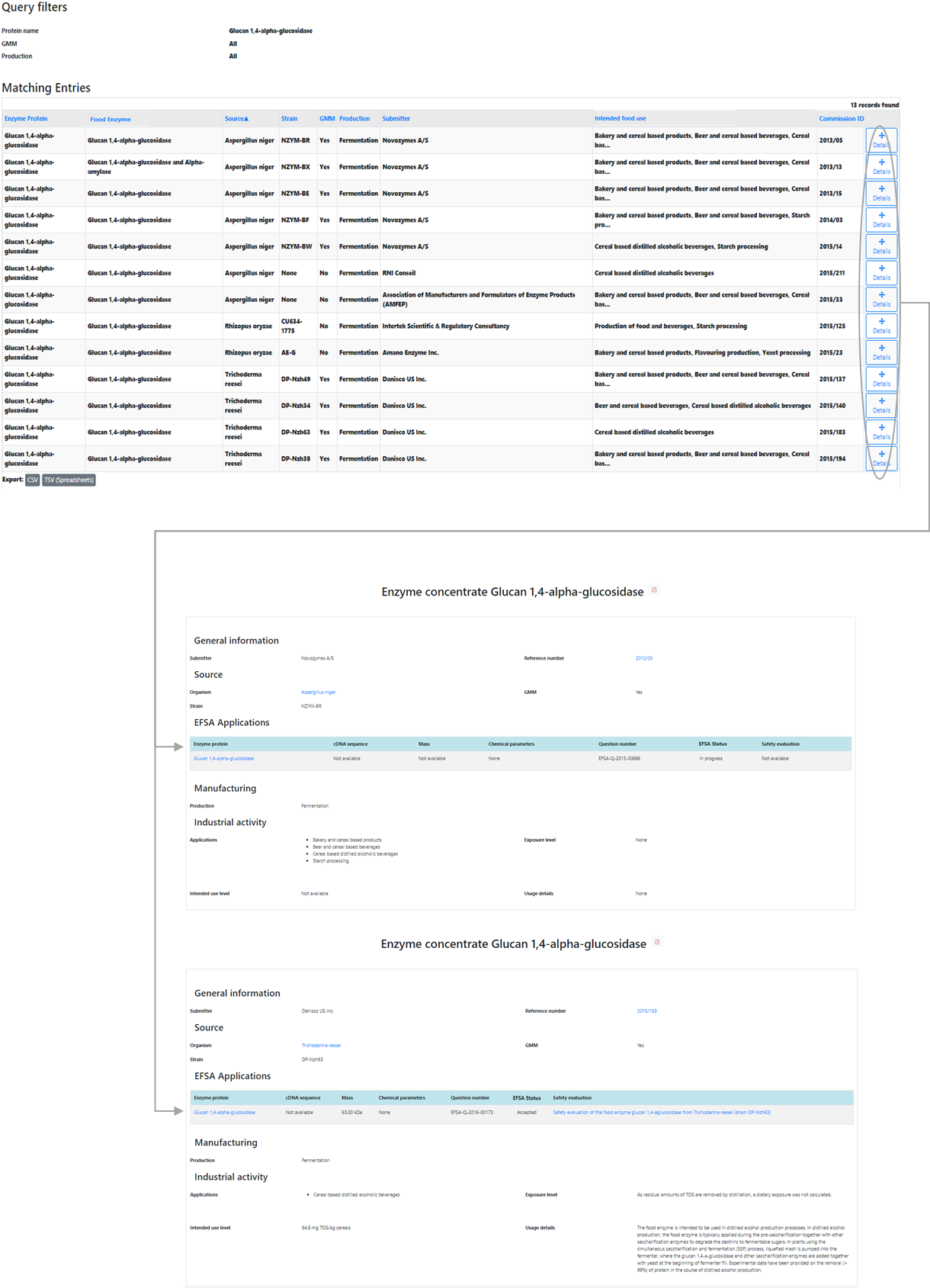


*Figure 4: Representation of the steps to follow in order to find the results of the study case 2 (Using the previous example of Glucan 1,4-alpha-glucosidase FE preparations, by inspecting the individual 13 FE pages, it can be observed that currently three FE have positively been evaluated by EFSA). By accessing the detailed pages from the search results, one can access the information about the EFSA evaluation status.*
